# Supplementary figures and images for: Tegoprazan–Amoxicillin Dual Therapy for Clarithromycin-Resistant Helicobacter pylori: A Feasibility Pilot Study
Source: Microorganisms. 2025 Oct 21;13(10):2408. doi: 10.3390/microorganisms13102408 (PMC12566439; doi:10.3390/microorganisms13102408)

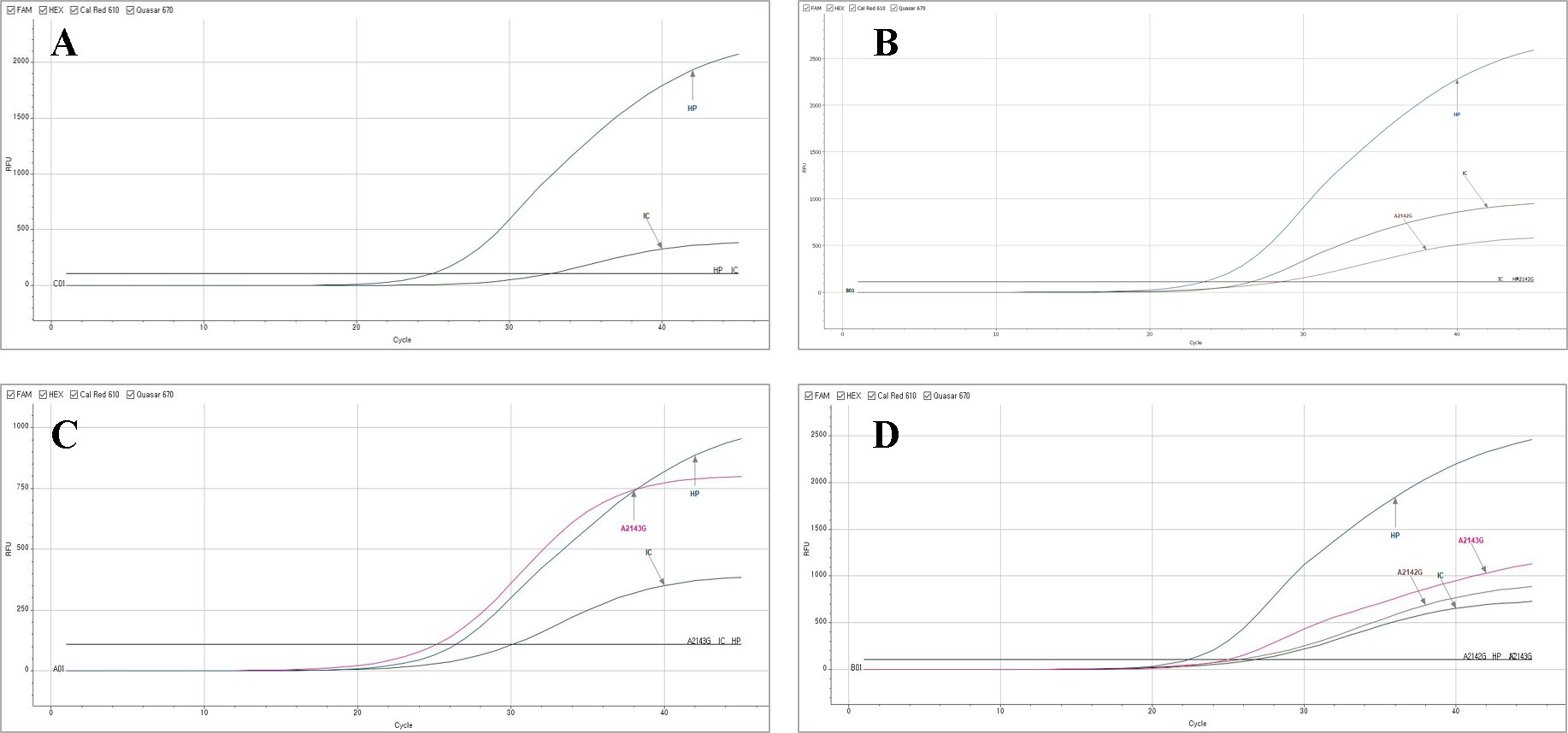

Supplement: Supplementary file 1 [file microorganisms-13-02408-s001.zip › Figure S1.tif]
